# Impact of Mistletoe Triterpene Acids on the Uptake of Mistletoe Lectin by Cultured Tumor Cells

**DOI:** 10.1371/journal.pone.0153825

**Published:** 2016-04-18

**Authors:** Katharina Mulsow, Thomas Enzlein, Catharina Delebinski, Sebastian Jaeger, Georg Seifert, Matthias F. Melzig

**Affiliations:** 1 Institute of Pharmacy, Department of Pharmaceutical Biology, Freie Universitaet Berlin, Berlin, Germany; 2 Department of Biotechnology, Mannheim University of Applied Sciences, Mannheim, Germany; 3 Department of Paediatrics, Division of Oncology / Haematology, Otto Heubner Centre for Paediatric and Adolescent Medicine (OHC), Charité Universitaetsmedizin Berlin, Berlin, Germany; 4 Birken AG, Niefern-Oeschelbronn, Germany; Jadavpur University, INDIA

## Abstract

Complementary treatment possibilities for the therapy of cancer are increasing in demand due to the severe side effects of the standard cytostatics used in the first-line therapy. A common approach as a complementary treatment is the use of aqueous extracts of Viscum album L. (Santalaceace). The therapeutic activity of these extracts is attributed to Mistletoe lectins which are Ribosome-inactivating proteins type II. Besides these main constituents the extract of Viscum album L. comprises also a mixture of lipophilic ingredients like triterpene acids of the oleanane, lupane and ursane type. However, these constituents are not contained in commercially available aqueous extracts due to their high lipophilicity and insolubility in aqueous extraction media. To understand the impact of the extract ingredients in cancer therapy, the intracellular uptake of the mistletoe lectin I (ML) by cultured tumor cells was investigated in relation to the mistletoe triterpene acids, mainly oleanolic acid. Firstly, these hydrophobic triterpene acids were solubilized using cyclodextrins (“TT” extract). Afterwards, the uptake of either single compounds (isolated ML and the aqueous “viscum” extract) or in combination with the TT extract (ML+TT, viscumTT), was analyzed. The uptake of ML was studied inTHP-1-, HL-60-, 143B- and Ewing TC-71-cells and determined after 30, 60 and 120 minutes by an enzyme linked immunosorbent assay which quantifies the A-chain of the hololectin. It could be shown that the intracellular uptake after 120 minutes amounted to 20% in all cell lines after incubation with viscumTT. The studies further revealed that the uptake in THP-1-, HL-60- and Ewing TC-71-cells was independent of the addition of TT extract. Interestingly, the uptake of ML by 143B-cells could only be measured after addition of triterpenes pointing to resistance to mistletoe lectin.

## Introduction

Cancer is the second most common cause of death after diseases of the cardiovascular system [1]. Since conventional chemotherapy has severe side effects, the demand for alternative treatments is obvious. One possibility is the additional use of herbal preparations of the European white berry mistletoe, *Viscum album* L. (Santalaceae) [2, 3]. These preparations usually consist of aqueos mistletoe extracts in which Mistletoe lectin I (ML), II, III, viscotoxins, polysaccharides and flavonoids are the principal active compounds. The main cytotoxic effect is induced by the mistletoe lectins I-III and based on its N-glycosidase-activity [4–7]. They are glycoproteins belonging to the ribosome-inactivating proteins (RIP) type II [8]. The anti-tumor and immunological effect has been demonstrated in different *in vivo* studies with acute myeloid leukemia, acute lymphoblastic leukemia, pancreatic cancer and melanoma [9–17]. However, the synergistic effects of the individual components are not properly clarified [18].

The uptake mechanism is similar to the uptake of other RIP type II, for example Ricin from *Ricinus communis* L. (Euphorbiaceae) [19–22]. The ML B-chain binds to cell membrane receptors with carbohydrate structures, especially to D-galactose groups [23–25], in the periphery of the cells including lamellapodia and cell contact regions [21]. It is absorbed by endocytosis mediated by clathrin-dependent or clathrin-independent mechanisms [21, 26]. After ingestion, ML is located in tubular structures, separated vesicles and in endosomal compartments as well [21, 27]. The ML A-chain acts as a toxin by inactivating the ribosome.

Besides the hydrophilic constituents, *Viscum album* L. contains also relevant lipophilic compounds, mainly triterpene acids (TT), like oleanolic (OA), betulinic and ursolic acid. The mentioned TT have anti-cancer effects [28, 29]. Furthermore, they induce anti-inflammatory and apoptotic effects as well as inhibition of cell growth [30–32]. Due to its structure, triterpenes are also able to embed in membranes and to disturb their structure [33–37] thereby facilitating the migration of ML into the cytoplasm.

Unfortunately, these compounds are not contained in the commercially available aqueous mistletoe extracts due to their high lipophilicity [38]. However, in classical phytomedicine it is frequently found that an extract is more effective than isolated single components [39–41]. Furthermore, the concomitant treatment with ML and TT has already shown a therapeutic effect *in vitro* and *in vivo* [42, 43]. The combined application of both substance classes becomes feasible by solubilization of the TT using 2-hydroxypropyl-β-cyclodextrin (2-HP-β-CD) [42, 44].

The aim of the current study was to examine the ML uptake by two leukemia and two sarcoma cell lines. The isolated ML as well as the aqueous extract (viscum) were tested either as single compound or combined with the TT containing extract (viscumTT). An enzyme linked immunosorbent assay (ELISA) served as method for determining the uptake of ML into the cells.

## Materials and Methods

### Extracts

The investigated mistletoe extracts were produced by Birken AG (Niefern-Öschelbronn, Germany). Sprouts from *Viscum album* L. were harvested from apple trees (*Malus domestica* Borkh., Rosaceae) and identified by the co-author S. Jaeger. To gain the viscum extract containing ML, the plant material was freeze-milled and afterwards extracted using ascorbate phosphate buffer. The detailed preparation has already been described [42]. ML was quantified using ELISA (n ≥ 3). The same plant material was used to obtain the TT extract by accelerated solvent extraction with n-Heptane. This method is also already published by Delebinski et al. (2015) [13]. The TT extract was solubilized by using 2-HP-β-CD and OA was quantified using GC-FID and external calibration with OA as reference substance (n ≥ 3) [45]. The three used viscum and TT extract batches are listed in Table 1. The extract preparation batches differed according to the used collection of plant material (June or July 2009 for viscum extract batches, May 2001 or May 2013 for TT extract batches) and to the used extract amount (3 or 4 mg). Various batches are used to prevent bias caused by a particular batch.

**Table 1:** Summary of the viscum and TT extract batches.

batch	plant material	viscum extract [mg mistletoe / mL extract]	buffer	2-HP-β-CD [mg/mL]	OA [mg/mL]	ML [μg/mL]
**viscum 155**	One-year-old shoots of mistletoe from apple trees harvest 03.06.2009	3 mg/g	30 mM phosphate pH 7.6 with ascorbic acid and sucrose	-	-	1.17 ± 0.17
**TT 155**	Mistletoe dry extract harvest 09.05.2011	-	60 mM phosphate pH 7.6 with sucrose	250	3.31 ± 0.08	-
**viscum 157L**	One-year-old shoots of mistletoe from apple trees harvest 03.06.2009	4 mg/g	30 mM phosphate pH 7.5 with ascorbic acid	-	-	1.67 ± 0.32
**TT 157L**	Mistletoe dry extract harvest 09.05.2011	-	60 mM phosphate pH 7.5	230	2.90 ± 0.09	-
**viscum 161**	One-year-old shoots of mistletoe from apple trees harvest 03.07.2009	3 mg/g	30 mM phosphate pH 7.5 with ascorbic acid and sucrose	-	-	2.19 ± 0.11
**TT 161**	Mistletoe dry extract harvest 23.05.2013	-	60 mM phosphate pH 7.5 with sucrose	240	3.60 ± 0.06	-

ML (mistletoe lectin I) was quantified using ELISA (n ≥ 3). OA (oleanolic acid) was quantified in each solution using GC-FID and external calibration with OA as reference substance (n ≥ 3) [45]. Values are mean ± SD.

The values of the ML uptake from the viscum and viscumTT experiments were calculated by using the following listed three viscum and TT extract batches, respectively in two different concentrations (Table 2).

**Table 2:** The used viscum and TT extract concentrations.

Batch	ML [ng/mL]	OA [μg/mL]
**viscum 155**	29	-
	22	-
**viscumTT 155**	29	20
	29	29
	22	20
	22	29
**viscum 157**	42	-
	31.5	-
**viscumTT 157**	42	18
	42	25
	31.5	18
	31.5	25
**viscum 161**	55	-
	41	-
**viscumTT 161**	55	25
	55	35
	41	25
	41	35

ML (mistletoe lectin), OA (oleanolic acid)

### Reagents and Media

The isolated ML was provided by Dr. U. Pfueller (Universitaetsklinikum Hamburg-Eppendorf, Germany, Department of Anatomy and Experimental Morphology). The ML (A-chain) antibodies (5F5 and 5H8, POD labelled) were purchased from Sifin diagnostics GmbH (Berlin, Germany). The cell media RPMI 1640 and DMEM, fetal calf serum, L-alanyl-L-glutamine-solution, Phosphate Buffered Saline (PBS) and Trypsin/EDTA-solution (0.05% / 0.02%) were purchased from Biochrom AG (Berlin, Germany). The medium IMDM was obtained from Lonza Cologne GmbH (Cologne, Germany). 3,3’,5,5’-tetramethylbenzidine was purchased from eBioscience Inc. (Frankfurt a.M., Germany). StabilZyme^®^ was obtained from Diarect AG (Freiburg, Germany). APC Annexin V and Propidium Iodide (PI) Staining Solution were purchased from BD Pharmingen (Heidelberg, Germany). All other chemicals were purchased from Sigma-Aldrich (Steinheim, Germany).

The lysis buffer consists of 150 mM sodium chloride, 1% Triton X-100 and 50 mM Tris at pH 8.0. The bicarbonate puffer pH 9.6 consists of 0.04 M sodium carbonate and 0.06 M sodium hydrogen carbonate. The binding buffer (pH 7.4) consists of 10 mM HEPES/NaOH, 140 mM sodium chloride and 5 mM calcium chloride.

### Cell Culture

The THP-1 cell line is a monocytic leukemia cell line and was purchased from the German Collection of Microorganism and Cell Cultures (DSMZ, Braunschweig, Germany). The cell line was cultivated as recommended in RPMI 1640 with supplements and subcultivated every three to four days. The human myeloid leukemia cell line HL-60 was obtained from the DSMZ (Braunschweig, Germany). The cultivation was carried out in RPMI 1640 with supplements according to the specifications. For the ELISA both cell lines were plated at 2.4 x 10^5^ cells/well into 24-well plates, final volume 400 μL. The cells were incubated for 24 h at 37°C with 5% CO_2_.

The 143B cell line is a human osteosarcoma cell line and was purchased from the American Tissue Culture Collection (ATCC, Wesel, Germany). The cells were maintained as recommended in DMEM with supplements. The Ewing’s sarcoma cell line TC-71 was obtained from DSMZ (Braunschweig, Germany) and was cultivated as recommended every two to three days in IMDM with supplements. For the ELISA the adherent cell lines were seeded at 1.2 x 10^5^ cells/well into 24-well plates and incubated as well for 24 h at 37°C with 5% CO_2_.

### Test Procedure

Cells were seeded and cultured at 37°C in saturated water vapor atmosphere with 5% CO_2_ and after 24 h incubated with either the isolated ML or three different viscum extract batches (each with and without the TT extracts) for 30, 60 or 120 min.

The ML concentrations were between 22–55 ng/mL and the TT concentration ranged (calculated as OA) from 18–35 μg/mL (Table 2). The used isolated ML concentrations—alone and in combination with TT 155—are shown in Table 3.

**Table 3:** Used isolated ML and isolated ML + OA concentrations.

Sample	ML [ng/mL]	OA [μg/mL]
**isolated ML**	66	-
	44	-
**isolated ML + TT 155**	44	20
	44	29
	33	20
	33	29

ML (mistletoe lectin), OA (oleanolic acid), TT (triterpene acid)

A separate standard series for the ELISA with isolated ML was generated for each plate and incubation time. After different incubation times, the content of ML in cells and supernatant was determined. The suspension cells were separated from the supernatant by centrifugation. Afterwards, a volume of 500 μL lysis buffer was added and the samples were shaken at 500 rpm at 4°C for 30 minutes. The adherent cells were washed with 200 μL PBS and were detached with 100 μL Trypsin/EDTA-solution. A volume of 400 μL lysis buffer was added and the samples were shaken at 500 rpm at 4°C for 30 minutes. All samples were centrifuged for 20 minutes at 4°C and 10900 rpm. The resulting solutions and the supernatants were subjected to ELISA.

### Enzyme Linked Immunosorbent Assay

The A-chain of the hololectin was determined with a modified sandwich ELISA [46–48]. The method is published by Delebinski et al. (2015) [13] and was modified only at the coating step where each well was coated with 100 μL capture antibody anti-ML 5F5 (0.9 μg in 1 mL bicarbonate buffer) instead of asialofetuin type I. The uptake by leukemia cells was tested for each single compound and for each combination in quadruplicate and the uptake by the sarcoma cells in quintuple.

### Flow Cytometric Analysis

To control the cell viability, PI and Annexin V binding assay were performed. Cells were cultivated at the same density for 24 h. Afterwards, the isolated ML and the three viscum extracts were added both with and without TT extracts for the same indicated time points. Cells were stained as described before [42]. The PI and Annexin V binding assay was performed for each sample and for each cell line in duplicate. Results were analyzed with FlowJo Software (TreeStar, Ashland, USA).

### Statistical Analyses

Dean-Dixon and Nalimov test were applied to determine outlier. Wilcoxon-Mann-Whitney was used to determine significant differences between treated cells and controls. The significance level was set at p ≤ 0.05 for all tests.

## Results

### Cell Viability

**Fig 1:**
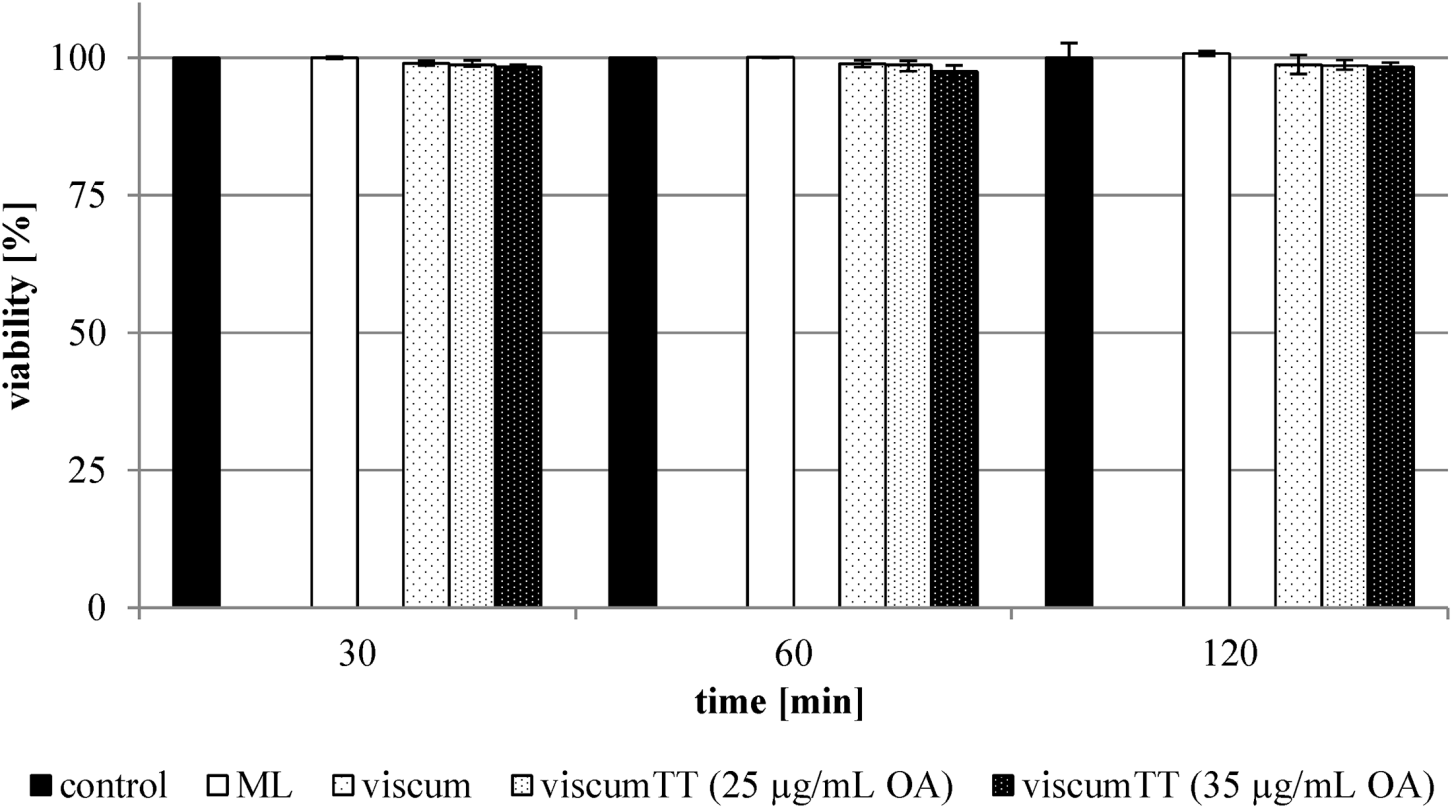
Cell viability of HL-60-cells. The cells were treated with different ML concentrations (see Tables 2 and 3) for 30, 60 and 120 minutes. The isolated ML, three viscum extract batches and the viscum extract batch 161 in combination with TT 161 extract batch (25 μg/mL and 35 μg/mL OA) were used. The viability was determined with Annexin V-APC and propidium iodide by flow cytometry. The values are expressed as percentages of the untreated control cells. Error bars represent the standard deviation of n = 2 experiments.

The PI and Annexin V binding assays displayed no significant differences between treated and untreated cells. Control cells had an average cell viability of 100 ± 6.7% and treated cells of 94.1 ± 10.7%. Fig 1 shows the cell viability for the HL-60-cellline.

### Mistletoe Lectin Uptake

After 120 minutes, an uptake of ML out of the viscumTT extract by all cell lines was observed (Table 4).

**Table 4:** Intracellular uptake of ML after incubation with viscumTT extract by different cell lines.

time [min]	ML-uptake by THP-1 [%]	ML-uptake by HL-60 [%]	ML-uptake by 143B [%]	ML-uptake by TC-71 [%]
**30**	7.2 ± 3.2	3.8 ± 1.9	3.3 ± 1.5	0.0 ± 0.0
**60**	12.4 ± 3.0	27.3 ± 9.7	1.1 ± 0.1	7.1 ± 6.2
**120**	12.6 ± 2.1	26.0 ± 0.2	19.9 ± 7.3	26.3 ± 0.9

The averages (± standard deviation) of the uptake were calculated using three viscumTT extract batches in two different ML (mistletoe lectin I) concentrations. For TT extract batches, the higher OA (oleanolic acid) concentration was used (TT 155: 29 μg/mL, TT 157: 25 μg/mL, TT 161: 35 μg/mL). The results are expressed as percentage of respectively used concentration (n ≥ 4).

The uptake by the THP-1 cells was the lowest of all cell lines. A saturation of ingestion occurred already after 60 minutes in the suspension cell lines THP-1 and HL-60. A considerable uptake by the adherent cell lines was detected after 120 minutes.

**Fig 2:**
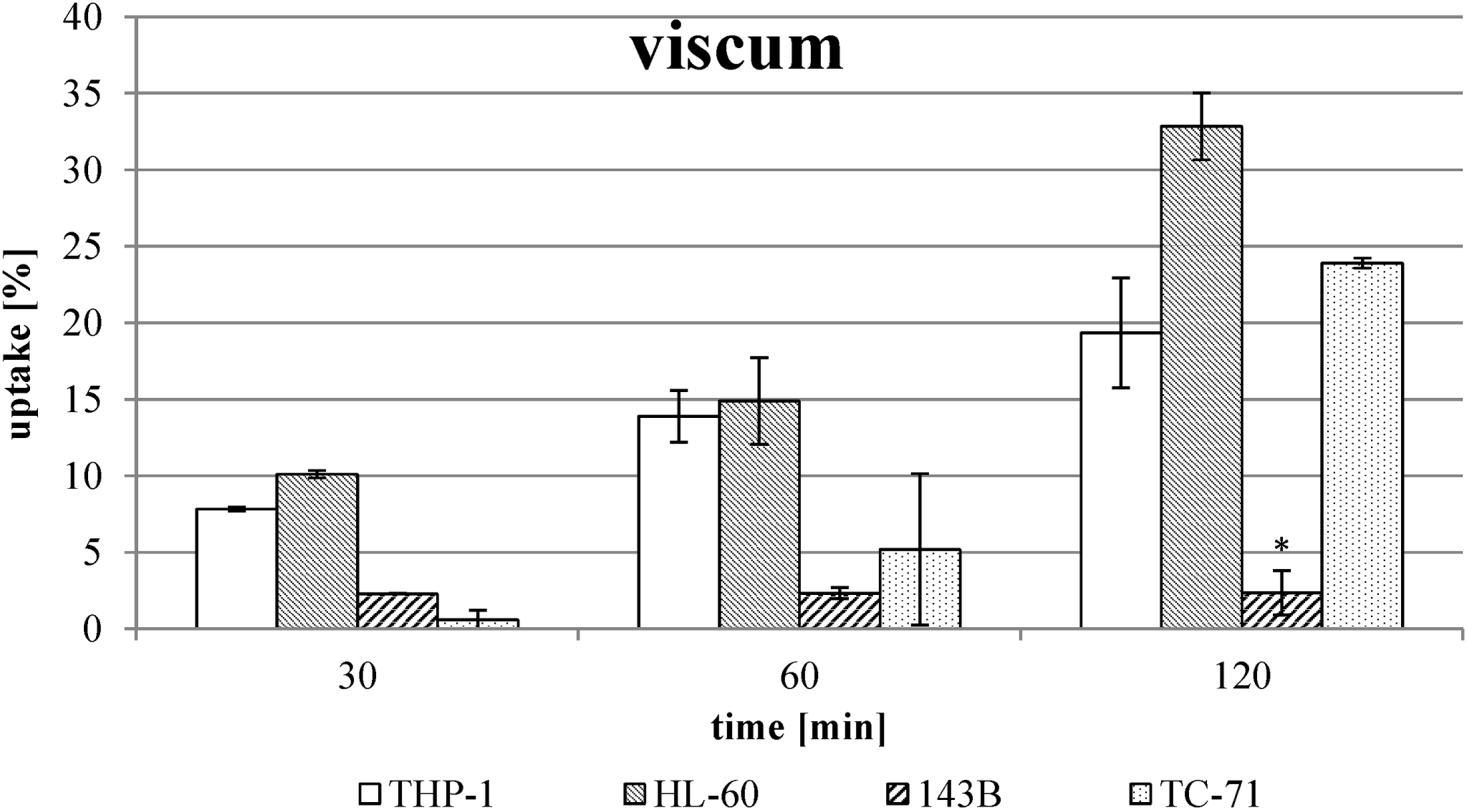
Uptake of ML out of viscum extract without combination with TT extract. The values were calculated using three viscum extract batches in two different ML concentrations (see Table 2) and are expressed as percentage of respective used concentration. Error bars represent the standard deviation of n ≥ 4 experiments. * Significant difference to the other cell lines, α ≤ 0.05.

The examination of the ML uptake out of the viscum extract without TT extract revealed no uptake by the osteosarcoma cell line 143B in contrast to the other cell models (Fig 2).

**Fig 3:**
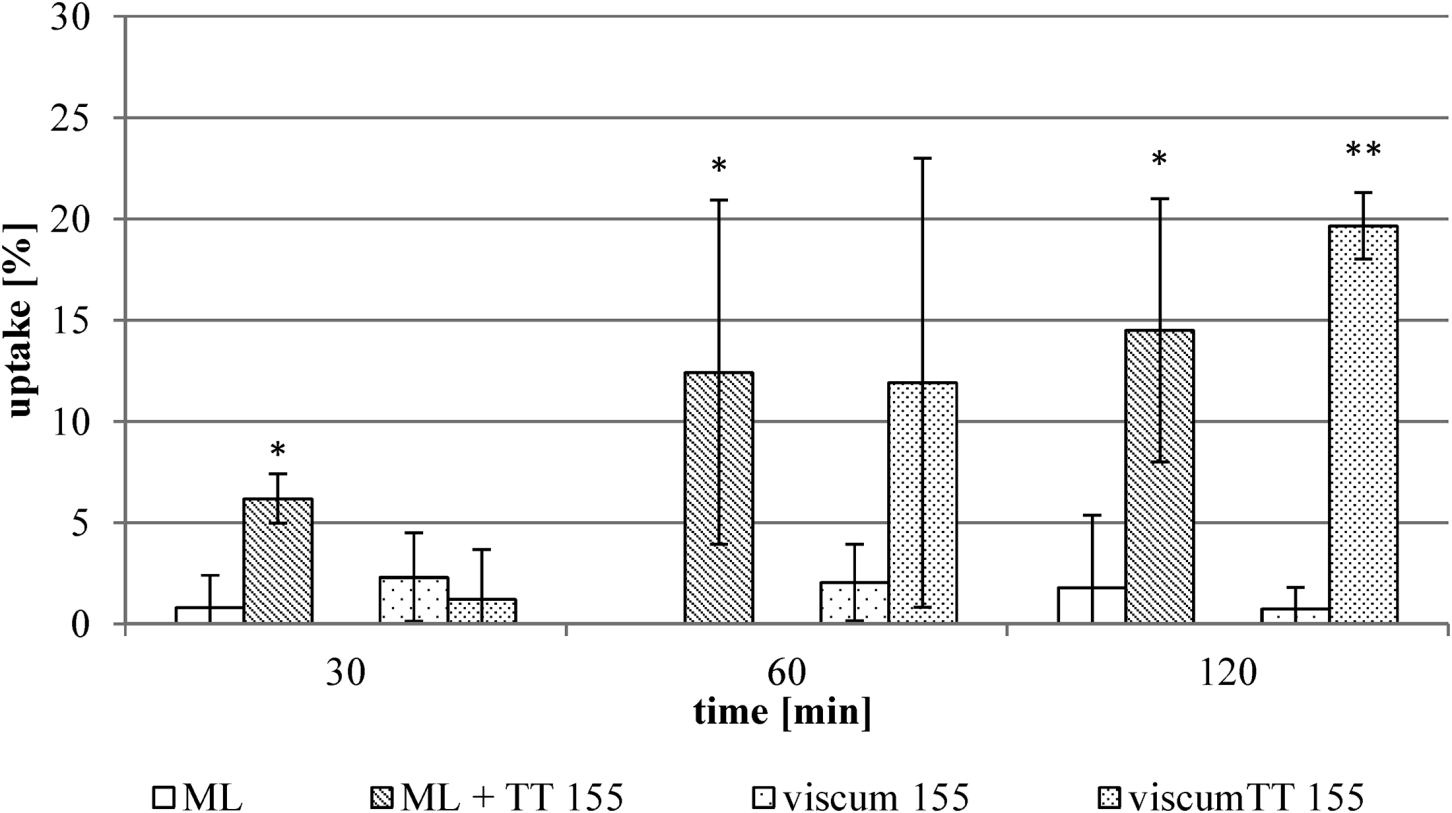
ML uptake by 143B cells. The averages of the uptake were calculated using isolated ML and viscum 155 extract batch in two different ML concentrations alone and in combination with TT 155 extract batch (20 μg/mL OA) (see Tables 2 and 3). The results are expressed as percentage of the respectively used concentration ± standard deviation of n = 5 experiments. * Significant difference to the isolated ML, α ≤ 0.05. ** Significant difference to the viscum 155 extract, α ≤ 0.05.

**Fig 4:**
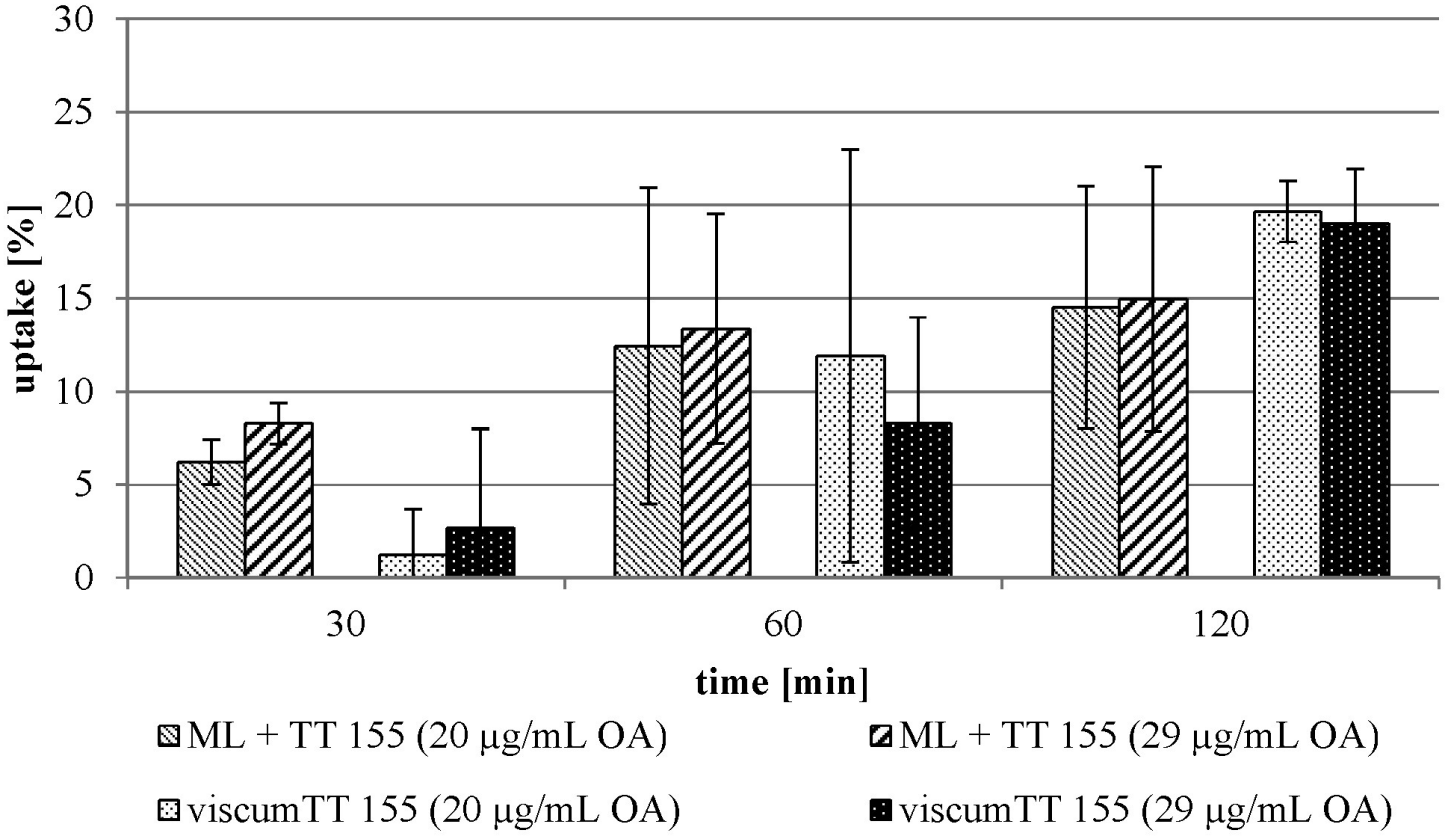
The TT concentration influence on the uptake by the 143B cells. The averages were determined using isolated ML and viscum 155 extract batch in combination with TT 155 extract batch (see Tables 2 and 3) and are expressed as percentage of the respectively used concentration. Error bars represent the standard deviation of n = 5 experiments.

**Fig 5:**
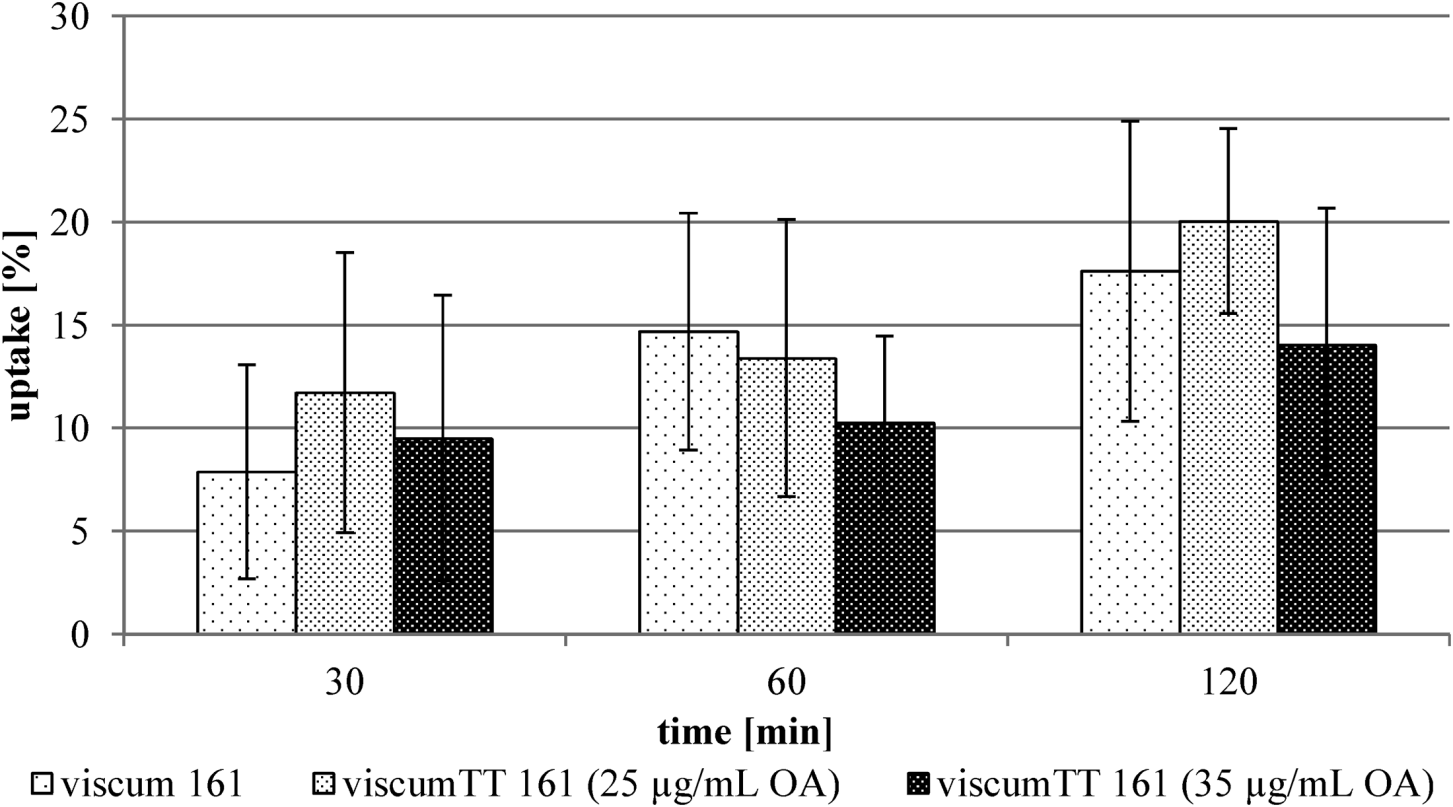
ML uptake out of viscum extract by the THP-1 cell line. The values were calculated using viscum 161 extract batch in two different ML concentrations alone and in combination with TT 161 extract batch (25 μg/mL and 35 μg/mL OA) (see Table 2). The results are expressed as percentage of respective used concentration. Error bars represent the standard deviation of n = 4 experiments.

In contrast to this finding, an uptake of ML by 143B cells was facilitated by combining the viscum and TT extract (viscumTT) (Fig 3). The uptake of the isolated ML increased from 1.8 ± 3.6% to 15.0 ± 7.1%. The uptake from the viscumTT extract was determined to be 20.2 ± 5.1%, in comparison with 2.3 ± 1.5% without TT extract. The different TT concentrations had no influence on the uptake (Fig 4). Surprisingly, the viscumTT extract did not alter the ML uptake by the other cultured tumor cell lines (exemplarily shown for THP-1 cell line, Fig 5).

2-HP-β-CD was used to solubilize the TT. The influence of the cyclodextrins on the uptake of ML was investigated in a separate experimental series with the 143B cell line (isolated ML, viscum extract, n = 3). The uptake of the ML was examined depending on the addition of 2-HP-β-CD. The addition of mere 2-HP-β-CD had no influence on the ML uptake (Table 5). After 120 minutes of incubation time, a significant difference between the addition of 2-HP-β-CD and the addition of TT extract could be observed.

**Table 5:** Comparison between 2-HP-β-CD and TT extract.

	ML-uptake [%]					
time [min]	ML	ML + TT	ML + CD	viscum 161	viscumTT 161	viscum 161 + CD
**30**	0.8 ± 1.6	6.2 ± 1.2	4.1 ± 4.9	2.3 ± 2.8	5.0 ± 4.6	4.1 ± 4.4
**60**	0 ± 0	12.4 ± 8.5	0 ± 0	2.7 ± 3.3	1.0 ± 1.3	0 ± 0
**120**	1.8 ± 3.6*	14.5 ± 6.5*	3.6 ± 2.9	2.67 ± 2.1	13.1 ± 2.4**	4.2 ± 0

The averages of the isolated ML (mistletoe lectin I) and of the ML + TT were determined using isolated ML in two different ML concentrations alone and in combination with TT 155 extract batch (20 μg/mL OA (oleanolic acid)) in quintuple. The combination ML + CD was tested in triplicate with 240 mg/mL 2-HP-β-CD (CD). The values of viscum 161 and viscumTT 161 were summarized using viscum 161 extract batch in two different ML concentrations and in combination with TT 161 extract batch (35 μg/mL OA) (n = 5). The combination viscum 161 + CD was tested in triplicate with viscum 161 extract batch and 240 mg/mL 2-HP-β-CD. The results are expressed as percentage of the respectively used concentration ± standard deviation.

* Significant difference to the isolated ML and to the combination ML + CD, α ≤ 0.05.

** Significant difference to the viscum 161 extract batch and to the combination viscum 161 + CD, α ≤ 0.05.

Furthermore, the isolated ML and the viscum extract were also examined with addition of a non-solubilized lipophilic extract (without 2-HP-β-CD). Instead of solubilizing, the lipophilic dry extract powder was solved in methanol (TT MeOH) and added at the same concentrations like the solubilized lipophilic TT extract before.

The addition of TT MeOH extract also increased the uptake of the ML after 120 minutes. However, the increase was not significantly higher when compared with the addition of the solubilized TT extract (Table 6).

**Table 6:** Comparison between TT extract solubilized with 2-HP-β-CD and solved in MeOH.

	ML-uptake [%]					
time [min]	ML	ML + TT	ML + TT (MeOH)	viscum 161	viscumTT 161	viscum 161 + TT (MeOH)
**30**	0.8 ± 1.6	6.2 ± 1,2	1.6 ± 2.1	2.3 ± 2.8	5.0 ± 4.6	1.5 ± 1.8
**60**	0 ± 0	12.4 ± 8.5	5.3 ± 4.4	2.7 ± 3.3	1.0 ± 1.3	4.3 ± 1.6
**120**	1.8 ± 3.6*	14.5 ± 6.5	18.7 ± 4.5	2.67 ± 2.1**	13.1 ± 2.4	19.6 ± 13.9

The averages of the isolated ML (mistletoe lectin I) and of the ML + TT were determined using isolated ML in two different ML concentrations alone and in combination with TT 155 extract batch (20 μg/mL OA (oleanolic acid)) in quintuple. The combination ML + TT (MeOH) was tested in triplicate (21 μg/mL OA). The values of viscum and viscumTT were calculated using viscum 161 extract batch in two different ML concentrations and in combination with TT 161 extract batch (35 μg/mL OA) (n = 5). The combination viscum 161 + TT (MeOH) was tested in triplicate (35 μg/mL OA). The results are expressed as percentage of the respectively used concentration ± standard deviation.

* Significant difference to the combination ML + TT and ML + TT (MeOH), α ≤ 0.05.

** Significant difference to the combination viscumTT 161 and viscum 161 + TT (MeOH), α ≤ 0.05.

## Discussion

The ML concentrations used in this study were chosen with respect to the detection limit of the ELISA-method and should be tolerated by the cells. Cell survival was determined by flow cytometry using the same incubation times and concentrations of ML and TT like in the uptake studies. There was no marked reduction in survival rate of cells in the concentration range tested. This supports the reliability of the study. In general, the leukemia cell lines were less prone to cytotoxicity by isolated ML and viscum extracts (average survival 98.7 ± 13.5%) than sarcoma cell lines (average survival 90.7 ± 7.4%). This may indicate an increased responsiveness to ML by sarcoma cell lines. Furthermore, the ML uptake from the viscum extracts by THP-1-, HL-60- and TC-71 cells was higher than the uptake of the isolated ML. This leads to the hypothesis that the higher intracellular availability of ML mediated by the extract leads to increased cytotoxity [5, 6, 18]. Minor differences in the manufacturing process of viscum extracts were averaged by using three different batches and consequently had no impact on the uptake of ML. The addition of TT extracts had no influence on the uptake of ML by the studied leukemia and Ewing’s sarcoma cell lines. This suggests that the structure of the RIP ML itself is sufficient for its uptake and there are a lot of binding sites for ML on the cell membrane.

For the 143B osteosarcoma cell line, only the addition of TT resulted in a measurable intracellular ML concentration. In this case, the application of solubilized TT using 2-HP-β-CD [42, 44] mediated the ML uptake. 2-HP-β-CD on its own did not enhance the ML uptake. The addition of a non-solubilized lipophilic extract (without 2-HP-β-CD) indicated an intracellular ML concentration. Therefore, the impact of mistletoe TT on the uptake of ML by 143B-cells was confirmed.

One reason for this observation might be the absence of binding sites, mainly D-Galactose structures [23–25], on the cell surface of 143B-cells. ML is assumed to bind to cell membrane structures in the same manner as RIP type II whereas the bound lectins enter the cells by receptor-mediated endocytosis [19, 20, 22, 49]. The attachment of the B-chain to the cell surface is a requirement for the uptake of lectins [50]. If both extracts are combined (viscumTT), the TT part might enable an uptake that is independent of the presence of binding sites for the B-chain. Due to its structure, triterpenes are able to embed in membranes and to disturb their structure [33–37] thereby facilitating the migration of ML into the cytoplasm. The examination of the impact of the TT concentration (calculated as OA) on the uptake of ML has shown no higher uptake with increasing TT concentration. With regard to *in vivo* trials, the smallest effective concentration should be determined since the used TT concentrations allowed a minimal lower cell viability of about 90% (90.7 ± 7.4%).

In future studies the question should be addressed whether the TT extract has an own impact on cytotoxicity. The TT extract could for example increase the sensitivity of cells for cytostatic compounds, especially for ML, which could enhance the therapeutic value of ML. Furthermore, an observation over a longer period is needed to investigate the cytotoxic effects of the TT—ML combination.

## Supporting Information

S1 Dataset Minimal data set underlying the findings of Figs 1–5. (XLSX)
